# An Event-Related Potential Study on Differences Between Higher and Lower Easy of Learning Judgments: Evidence for the Ease-of-Processing Hypothesis

**DOI:** 10.3389/fpsyg.2022.779907

**Published:** 2022-03-18

**Authors:** Peiyao Cong, Ning Jia

**Affiliations:** Department of Education, Hebei Normal University, Shijiazhuang, China

**Keywords:** easy of learning judgment, event-related potentials, source analysis, encoding fluency, ease-of-processing hypothesis

## Abstract

Easy of learning (EOL) judgments occur before active learning begins, and it is a prediction of how difficult it will be to learn new material in future learning. This study compared the amplitude of event-related potential (ERP) components and brain activation regions between high and low EOL judgments by adopting ERPs with a classical EOL judgment paradigm, aiming to confirm the ease-of-processing hypothesis. The results showed that (1) the magnitudes of EOL judgments are affected by encoding fluency cues, and the judgment magnitude increases with encoding fluency; (2) low EOL judgments are associated with higher N400 amplitude at the left superior frontal gyrus (SFG) and left middle frontal gyrus (MFG). High EOL judgments showed enlarged slow-wave (600–1,000 ms) potentials than low EOL judgments at the left medial temporal lobe (MTL), right ventromedial prefrontal cortex (VMPFC), and dorsolateral prefrontal cortex (DLPFC). Our results support the ease-of-processing hypothesis, particularly, by affirming that EOL judgments are affected by encoding fluency in two processing stages. N400 reflects the process of acquiring encoding fluency cues, while slow-wave indicates that individuals use encoding fluency cues for metacognitive monitoring.

## Introduction

Metacognitive monitoring refers to one’s self-assessment of their performance in completed, ongoing, or upcoming cognitive activities (Bellon et al., 2020). Typical metacognitive monitoring forms include easy of learning (EOL) judgments, judgments of learning (JOL), feeling of knowing (FOK) judgments, and judgments of confidence (JOC). Previous studies have focused on the behavior and neural mechanisms of JOL (Yang et al., 2015; Müller et al., 2016), FOK (Chua et al., 2009; Irak et al., 2019), and JOC (Morales et al., 2018). Few studies have focused on EOL judgments, which occur before active learning begins to predict how difficult learning new materials could be (Jemstedt et al., 2017; Lemieux et al., 2019). EOL judgment magnitude refers to the subjective estimate of an individual’s judgment of the difficulty in a memory task and can reflect the confidence of individuals. Judgment magnitude is a primary issue in EOL judgment research (Wu, 2012). EOL judgment magnitude presumably guides students’ study decisions and initial learning strategies (Jemstedt et al., 2017) and helps in improving the ability of metacognitive monitoring, prompting learning efficiency. The ease-of-processing hypothesis, which was proposed by Undorf and Erdfelder (2011), posits that if one needs to spend more time on an item, it means the encoding of this item lacks fluency and the likelihood of recalling the item is low, and this is regarded as low metacognitive judgment. The ease-of-processing hypothesis explained that there exist differences between metacognitive monitoring magnitudes, emphasizing that encoding fluency as a memory cue has an impact on the degree of metacognitive monitoring. It is also claimed that encoding fluency is a cue to making EOL judgments (Jemstedt et al., 2018). Researchers have also attempted to explore the brain mechanism of EOL judgments. Rosen et al. (2014) conducted a study of frontotemporal dementia patients, and results showed that the accuracy of item-by-item EOL judgments in patients was low, and they often underestimated their memory performance. Based on the results, Rosen et al. (2014) speculated that the function of frontal-temporal regions is related to EOL judgments. However, surprisingly few studies have investigated the neural mechanism in EOL judgments and previous studies have not specified the neural mechanism of EOL judgments in different time windows, meaning it is quite difficult to provide strong evidence of how neural mechanisms impact behavior. The underlying neural basis in EOL judgments needs to explore. Previous studies have not specified the neural mechanism of EOL judgments in different time windows, meaning it is quite difficult to provide strong evidence of how neural mechanisms impact behavior. Therefore, this study is the first attempt to investigate the processing mechanism of EOL judgments by adopting event-related potentials (ERPs) technology to analyze brain wave performance in different time windows and determine the time process of EOL judgments. As an attempt, this study would like to reference the JOL analysis method to explore the neural mechanism of EOL judgments and use source analysis to explore brain regions corresponding to ERP components.

To determine the ERP components of EOL judgments, this study referred to the findings of existing studies on other types of metacognitive monitoring. This study chose JOL as a similar metacognitive monitoring type as EOL to determine the corresponding ERP components is based on the following two reasons. Firstly, metacognitive monitoring has many types, such as EOL, JOL, and JOC, which are essentially an assessment of individual confidence while occurring at different processing stages. EOL judgments and JOL occur before a test. However, JOC occurs after a test. According to these criteria, some researchers have divided metacognitive monitoring into prospective monitoring and retrospective monitoring. Prospective monitoring (EOL and JOL) refers to students’ judgments about how well they will perform on an item on a future test (Baars et al., 2014). Retrospective monitoring (JOC) refers to students’ assessments of how well they performed on an item just completed (Baars et al., 2014). Both EOL judgments and JOL belong to prospective metacognitive monitoring, and behavioral studies have found that EOL judgments and JOL may have an association. For example, Voloshyna and Jonsson (2012) used 40 Ukrainian and Swedish word pairs to explore the correlation between EOL judgments and JOL. It was found that there exists a high correlation between them. It can thus be speculated that these two metacognitive monitoring may follow the same cognitive process. Secondly, EOL judgments and JOL use encoding fluency clues to make judgment magnitude. For instance, EOL judgment magnitude would be affected by an individual’s belief and encoding fluency (Jemstedt et al., 2017). JOL magnitude relies upon more clues, such as encoding fluency and retrieval fluency clues (Jia, 2012). It can be seen that EOL judgments and JOL to some extent have similarities in behavioral aspects. Therefore, the ERP results of JOL would provide reference information for EOL judgments.

N400 is one of the components to be focused on in the EOL judgment study. Just as we previously mentioned, EOL judgments have a correlation with fluency. N400 is an index of fluency in a metacognitive monitoring study. For example, Undorf et al. (2020) recorded ERPs while participants studied related and unrelated word pairs in the JOL task. The results showed the unrelated word pairs with lower JOL magnitudes and a higher N400 amplitude in midline electrodes than related word pairs. In their experiment, unrelated word pairs lack fluency and need deep processing than related word pairs, thus would produce higher N400 amplitude. Based on these results, the researchers concluded that fluency, as indexed by N400 amplitude, contributed to JOL. Besides, previous studies have reported that low JOL induced stronger positive slow-wave potentials in the frontal region and enlarged negative slow-wave potentials in the parietal region, from 350 to 800 ms (Müller et al., 2016), confirming that attempt retrieval cues were used to make JOL and reflected monitoring after retrieval. Thus, this study focuses on the N400 component and slow-wave potentials (600–1,000 ms).

Moreover, according to previous research of JOL, N400 and slow-wave components were significantly different in high JOL and low JOL. Hence, it can be inferred that there are two-time processing stages in JOL. It has been demonstrated that N400 can be used to obtain encoding fluency cues, while slow-wave potentials can use encoding fluency cues to produce judgment magnitudes. By the analogy of the brain mechanism of JOL, this study adopted ERP technology to explore the differences of EOL judgment magnitudes at different time windows on the ERP amplitude. It then analyzed the influence of encoding fluency on EOL judgment magnitudes and verified the ease-of-processing hypothesis through ERP analysis and source analysis. Based on the findings of previous literature, this study puts forward the first hypothesize: high EOL judgments and low EOL judgments post different brain waves in ERP mean amplitude at different time windows.

The neural mechanism results from the JOL task would provide valuable evidence for investigating EOL judgments’ brain regions. Previous studies have explored a large number of brain regions related to the JOL task, including the prefrontal cortex, temporal cortex, occipital cortex, and angular gyrus (Cosentino et al., 2015; Yang et al., 2015; Hu et al., 2017; Tsalas et al., 2018; Gaynor and Chua, 2019; Irak et al., 2019, 2020; d’Oleire Uquillas et al., 2020; Kelley et al., 2020; Undorf et al., 2020). Furthermore, Vaccaro and Fleming (2018) used a meta-analysis to find that prospective metacognitive monitoring involved the posterior medial prefrontal cortex, the left dorsolateral prefrontal cortex (DLPFC), the right inferior frontal gyrus, and the right insula, which would provide some evidence for EOL judgments. Both EOL and JOL are prospective metacognitive monitoring, and it could be speculated that they involved similar brain regions to some extent. Brain injury studies have also noted that the frontotemporal cortex is associated with EOL judgments (Rosen et al., 2014). During the process of EOL judgments, encoding fluency cues may activate coding-related brain regions and metacognitive monitoring-related brain regions. According to this logic, this study presents the second hypothesis: the distinction between high and low EOL judgments is related to the activation intensity of encoding brain regions and metacognitive monitoring brain regions.

## Materials and Methods

### Participants

Thirty-two undergraduate university students (16 men, 16 women; *M*_age_ = 22.21, *SD* = 2.58) from different faculties (e.g., education science, literature, history, and physics) were given informed consent and received appropriate financial compensation. All participants were right-handed Chinese speakers and reported normal or corrected-to-normal vision and no neurological or psychiatric disorders. This study was conducted following the approval of the local ethics committee, and participants were fully informed about the study purpose upon completion.

### Materials

The preparation process of materials is as follows: three hundred and forty Chinese character words in low word frequency from 0.01 to 8.63 were firstly selected from the Frequency Dictionary of Modern Chinese (Beijing Language and Culture University, 1986). Each word has two characters which are nouns. After random combination, 170 Chinese character word pairs were rated by thirty-nine undergraduate students using a six-point scale to give difficult degrees: 1 (very easy) and 6 (very hard). Finally, one hundred and twenty-five word pairs, which consisted of two Chinese characters, were chosen as formal experimental materials, including fifty-five easy word pairs (*M* = 5.10, *SD* = 0.64) and seventy difficult word pairs (*M* = 1.69, *SD* = 0.61). To ensure the homogeneity of materials in the experiment and eliminate the interference of irrelevant factors on the participants, word frequency and stroke number of easy and hard word pairs between cue words and target words were carried out the test of significance of the difference (see Supplementary Tables 5, 6 for more details). The results showed no significant difference in word frequency and stroke number of easy and hard word pairs between cue words and target words. Each word pair consisted of two Chinese characters words, such as “huai zhang — ye ma” (坏账—页码), the words on the left are cue words and the words on the right are target words (see Supplementary Table 1 for an example). All word pairs are divided into five groups, containing 10 easy word pairs and fifteen difficult word pairs.

### Design and Procedure

A single variable (EOL judgment conditions: high or low) within-participants design was used. High EOL judgment ratings (4, 5, and 6) and low EOL judgment ratings (1, 2, and 3) were calculated as high and low EOL judgment conditions. The dependent variables were recognition performance and the response time (RT) of EOL judgments, and the relative accuracy of EOL judgments was calculated using Goodman–Kruskal’s Gamma correlation (Nelson, 1996). ERP was measured as N400 and slow-wave mean amplitude.

**FIGURE 1:**
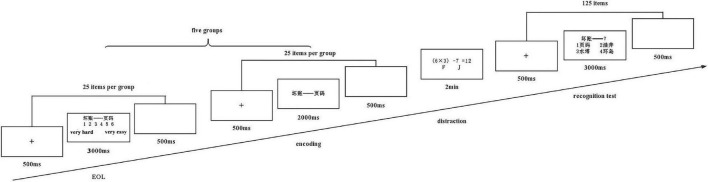
The procedures of the experiment were used in the study.

Before the experiment started, participants were fully informed of the purpose and the nature of it. The experiment was conducted using E-prime 2.0 software (E-Prime 2.0 Psychology Software Tools, Inc., Pittsburgh, PA, United States). The experimental procedure was completed in a shielded room, and all stimuli were presented with the same brightness on a Windows XP computer with a 21-inch monitor. The computer screen had a resolution of 1,920 × 1,080, and the eye-screen distance was approximately 60 cm. All word pairs were displayed in black 40-point Song typeface font on a white background. The task used in the current investigation is an EOL judgments’ paradigm, in accordance with Jemstedt et al. (2018) study, which included four consecutive phases: an EOL phase, a study phase, a distraction phase, and a recognition phase (see Figure 1). The EOL judgments informed participants to use a 6-point scale from 1 to 6 (1: very hard to learn; 6: very easy to learn) to make a judgment about the difficulty of studying each word pair. EOL judgment task and encoding task contain one hundred and twenty-five word pairs separately. Both were divided into five groups. The participants were presented with ten practice trials before the formal experiment to provide a relevant experience. When finishing the EOL judgments’ task, participants were instructed to complete the encoding task. Then a distraction task is presented after the encoding task has been done to inhibit the recitation from studied materials. Finally, participants completed a recognition test for which each cue word was presented alongside four alternatives that consisted of correct target words, and the other three alternatives were selected from studied word pairs.

An EOL judgment trial began with a fixation cross (duration: 500 ms), then one Chinese character word pair was presented, and participants were instructed to make EOL judgments for the maximum time limitation of 3 s. EOL judgment question is as follows: “How difficult will it be to learn the word pairs between the right and left Chinese character words?” This question was first answered in the EOL judgments’ task by using a computer keyboard. After typing in the EOL judgments, a white screen was shown for 500 ms. After finishing a part that included twenty-five trials, participants were required to finish the next study part.

A study trial also started with a fixation cross (duration: 500 ms). Next, a word pair was shown for 2 s, and participants were required to try their most to memorize it. The study trial ended up with a white screen whose duration was 500 ms.

After studying all word pairs of Chinese characters, arithmetic equations were presented that were instructed to judge as correct or incorrect by pressing the “F” or “J” key within 10 s, respectively. For example, for the equation “304 − 286 = 18,” participants had to press F to indicate that the solution was correct. This distractor task lasted for 180 s and served to prevent rehearsal of the study material.

A recognition test phase contained all word pairs participants have studied. A recognition trial was started with a fixation cross for 500 ms, then a cued item of two Chinese characters was presented on the screen with four alternatives, which contained one target item and three disturbance items. The target items were balanced. Simultaneously, participants were asked to choose the target item in 3 s, ending with a white screen for 500 ms as before.

### Electroencephalogram Recording and Preprocessing

The Electroencephalogram (EEG) was recorded during the EOL rating phase using a Neuroscan System according to the extended international 10–20 system (Picton et al., 2000) using 62 Ag/AgCI electrodes positioned in an elastic nylon cap. EEG signals were amplified and recorded at a 1,000-Hz sampling rate using a SynAmps2 amplifier. The EEG recordings were referenced to the left mastoid (M1). All EEG electrode impedance was kept below 5 KΩ. Vertical (vEOG) and horizontal electrooculograms (hEOG) were recorded as the voltage difference between the electrodes positioned above and below the left eye and between those to the left and right canthi of the eyes, respectively.

Offline analysis was conducted in MATLAB using the EEGLAB toolbox (Delorme and Makeig, 2004) and applied to each participant’s datasets. All signals were band-pass filtered (0.1–40 Hz) (Skavhaug et al., 2010), and re-referenced was based on the common average reference method (CAR). Independent component analysis (ICA) was performed to correct the components associated with eye movement and eye-blink artifacts. Then, segments containing values ± 80 μV were excluded using extreme value rejection (Skavhaug et al., 2010). In addition, data were visually inspected for artifacts that were missed by the automated procedure, and these artifacts were excluded from the analysis. Bad channels were replaced by an interpolated weighted average from surrounding electrodes using the EEGLAB toolbox in MATLAB (Delorme and Makeig, 2004). According to a metacognitive monitoring study (Irak et al., 2019), the EOL judgment phase was formed for two categories, high EOL judgment ratings (4, 5, 6) and low judgment ratings (1, 2, 3), and they were calculated as high and low EOL judgments. Then data that contained EOL judgment conditions (high, low) were extracted from − 200 to 1,000 ms (1,200 ms) following stimulus onset. The 200 ms before stimulus onset was defined as a prestimulus baseline. Segments were baseline corrected (–200 to 0 ms), and artifact-free segments for all responses were averaged separately for each participant and each condition (low/high). ERPs were exported as mean amplitudes per electrode within specific time windows for statistical analysis.

### Event-Related Potential Analysis

**FIGURE 2:**
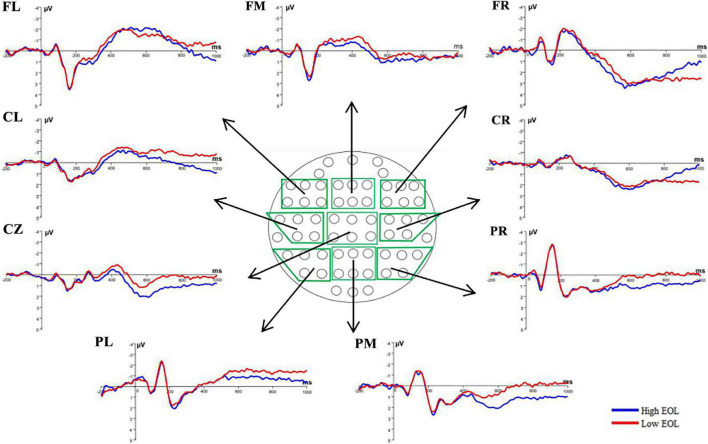
Stimulus-locked event-related potential (ERP) grand average (*N* = 32) in low (red line) and high (blue line) EOL judgment conditions is showed at one frontal-left electrode (F3), one frontal-middle electrode (Fz), one frontal-right electrode (F4), one central-left electrode (C3), one central-middle electrode (Cz), one central-right electrode (C4), one parietal-left electrode (P3), one parietal-middle electrode (Pz), and one parietal-right electrode (P4) were corrected as Stimulus-locked average ERP of each region of interest (ROI; *N* = 32) in low (red line) and high (blue line) EOL judgment conditions are showed at frontal-left (FL) ROI, frontal-middle (FM), frontal-right (FR), central-left (CL), central-middle (CM), central-right (CR), parietal-left (PL), parietal-middle (PM), and parietal-right (PR). The nine ROIs for ERP analysis are marked with green color in the middle head plot.

Our main analysis of averaged ERPs focuses on the N400 (400–600 ms) and slow-wave potentials (600–1,000 ms). Amplitude values of the ERPs of the participants were obtained by averaging the EEG values. ERP grand averages were calculated for two conditions: high EOL and low EOL judgments (see Figure 2). Based on a previous study (Yu et al., 2021), the amplitude of the N400 component was quantified as the mean amplitude from 400 to 600 ms. The mean amplitudes of slow-wave potentials were measured and compared in 100 ms step windows. There are three reasons for the measurement differences between N400 and slow-wave potentials. Firstly, the N400 is a negative ERP component that mainly occurs 400–600 ms after stimulus onset (Kutas and Federmeier, 2011; Undorf et al., 2020). N400 is a certain ERP component, and it would inevitably lose valuable information if we use 100 ms step windows to measure N400. Secondly, this study aims to use source analysis to explore brain regions in N400 time windows and slow-wave potentials. It would be possible to discover which brain regions function to produce N400 from 400 to 600 ms. Slow-wave potentials have no specific peak or clear component (Rösler et al., 1997), and using 100 ms step windows for ERP analysis and source localization would be cautious. Thirdly, this study referred to the ERP and source analysis of Yu et al. (2021), whose study focused on P3 and slow-wave components. They also use different time windows to measure the two components. For P3, they use a time window from 300 to 600 ms, while slow-wave potentials use 100 ms step windows. The N400 and slow-wave potentials were accessed among nine regions of interest (ROI) to assess the scalp distribution. The ROIs were frontal-left (FL): F3, F5, F7, FC3, FC5, FT7; frontal-middle (FM): F1, Fz, F2, FC1, FCz, FC2; frontal-right (FR): F4, F6, F8, FC4, FC6, FT8; central-left (CL): C3, C5, T7, CP3, CP5; central-middle (CM): C1, Cz, C2, CP1, CPz, CP2; central-right (CR): C4, C6, T8, CP4, CP6; parietal-left (PL): P3, P5, P7, PO3, PO5, PO7; parietal-middle (PM): P1, Pz, P2, PO3, POz, PO4; and parietal-right (PR): P4, P6, P8, PO4, PO6, PO8. The average number of usable segments of artifact-free was comparable in the high EOL judgment conditions (*M* = 52.22, *SD* = 12.79) and low EOL judgment conditions (*M* = 58.03, *SD* = 13.45). Three-factor repeated-measures ANOVAs with 2 (condition: high, low) × 3 (region: frontal, central, parietal) × 3 (hemisphere: left, middle, right) were performed on the mean amplitudes of the electrodes (in corresponding ROIs) in SPSS 23.0, applying the Greenhouse-Geisser correction (Greenhouse and Geisser, 1959) where appropriate. All reported differences are significant at the level of *p* < 0.05 and were followed by the Bonferroni *post hoc* comparison test if necessary (Tabachnick and Fidell, 2013).

### Source Analysis

Source analysis was performed with minimum norm estimate (MNE) (Gramfort et al., 2014) for grand averaged ERPs under different conditions and was done using FieldTrip software^1^. The basic theory of the MNE algorithm is to estimate the dipole matrix based on EEG data, assuming that a large number of dipoles are distributed in the brain. The dipole matrix is used to calculate the energy value (measured by L2 norm), and the solution with the least energy is obtained to estimate the source signal. MNE divides the human brain into several grids, and it is of 2,459 points with 1 cm spacing that was created in the template Montreal Neurological Institute (MNI) brain (Mazziotta et al., 2001), well-distributed in the cortex, which represents the source signals of EEG. This structure is close to the physical structure of the human brain. Next, the L2 norm is used to estimate the minimum current density to obtain the unique solution and reflect in the real head model (MNI152 template).

To analyze the intensity difference of activation between brain regions in conditions (high, low), the paired sample *t*-test was performed in N400 and slow-wave time intervals for the source activity brain regions of different conditions. Finally, a cluster permutation test (number of randomizations = 5,000) was performed to correct *t* values for multiple comparisons (Pernet et al., 2015). Brain regions with significant differences (*p* < 0.05) between conditions (high, low) were reported as Brodmann areas (BA) and the MNI coordinates.

## Results

### Behavioral Results

The behavioral analysis referring to Liu et al. (2019) calculated the Goodman–Kruskal’s Gamma correlation for each participant between EOL judgment conditions and recognition performance at first. A one-sample *t*-test was performed and revealed that Gamma correlations were more significant than 0 [*t*(31) = 11.81, *p* < 0.001], which showed EOL judgments of participants were not a random guess.

Then repeated-measure ANOVA was conducted for RT and recognition performance of each EOL judgment magnitude (see Supplementary Table 2 for more details). The main effect of EOL judgment RT on six EOL judgment magnitudes was significant, *F*(5, 155) = 19.85, *p* = 0.000, η^2^ = 0.39. The EOL judgment RT of magnitudes 3 and 4 were the longest, followed by magnitude 1, magnitude 2, and magnitude 5, the EOL judgment RT of magnitude 6 was the fastest. The main effect of recognition performance of six EOL judgment magnitudes was significant, *F*(5, 155) = 19.70, *p* = 0.000, η^2^ = 0.39. The recognition performance of EOL judgments was increased with magnitudes. Participants are likely to recognize an item that was previously judged easier to learn in the EOL phase, which would cause better recognition performance in the test. With the magnitude increase in the EOL judgment phase, items judged easier to learn that would have higher successful recognition outcomes in the last test.

Lastly, the paired sample *t*-test was used to compare high EOL judgments and low EOL judgments, and the results showed a significant difference in EOL judgment RT (ms) between high EOL and low EOL judgments [*t*(31) = 5.59, *p* < 0.001], with higher judgment RT for low EOL judgments than high EOL judgments (see Supplementary Table 3). EOL judgments’ judgment time (RT) decreased with EOL judgment magnitude rise.

The paired sample *t*-test revealed a significant difference in recognition performance between high EOL and low EOL judgments [*t*(31) = − 6.12, *p* < 0.001], with better performance for high EOL judgments than for low EOL judgments (see Supplementary Table 3). An item was judged as easy to learn in the EOL phase, and participants would perform better for this item in the recognition test. However, when an item was rated hard to learn, participants hardly recognized this item in the test.

### Event-Related Potential Results

**FIGURE 3:**
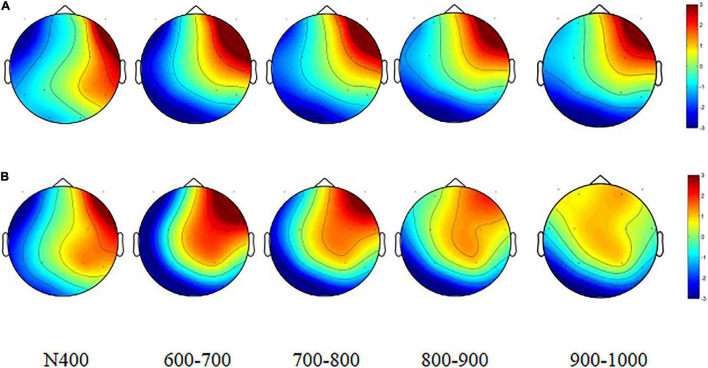
Head plots in low and high easy of learning (EOL) judgments. **(A)** Low EOL judgment head plots. **(B)** High EOL judgment head plots. Stimulation applied at “0.0 ms” time point (stimulus onset).

Figures 2, 3 show the grand average and scalp topographies filtered waveform during high EOL and low EOL judgment conditions for nine ROIs. Both the figures show that the prestimulus EEG serves as the baseline in the present study and the post-stimulus ERP. Supplementary Table 4 demonstrates the ERP results of 2 (condition: high, low) × 3 (region: frontal, central, parietal) × 3 (hemisphere: left, middle, right) repeated-measured ANOVA.

### Analysis of Average Event-Related Potentials: N400 (400–600 ms)

A 2 (condition) × 3 (region) × 3 (hemisphere) repeated-measures ANOVA revealed a main effect for condition, *F*(1, 31) = 13.22, *p* < 0.001, η^2^ = 0.30. N400 yielded significant interaction effects for condition × hemisphere, *F*(2, 62) = 5.06, *p* < 0.001, η^2^ = 0.14. Simple effect analysis revealed that in middle ROIs, low EOL judgment conditions were higher in amplitude than high EOL judgments (*p* < 0.001).

### Analysis of Average Event-Related Potentials: Slow-Wave Potentials (600–1,000 ms)

For the ERP slow-wave potentials from 600 to 700, 700–800, 800–900, and 900–1,000 ms, all the ANOVAs revealed significant main effect for condition, *F*_600–700_ (1, 31) = 16.29, *p*_600–700_ < 0.001, η^2^_600–700_ = 0.34; *F*_700–800_ (1, 31) = 12.10, *p*_700–800_ < 0.001, η^2^_700–800_ = 0.28; *F*_800–900_ (1, 31) = 16.44, *p*_800–900_ < 0.001, η^2^_800–900_ = 0.35; *F*_900–1,000_ (1, 31) = 11.80, *p*_900–1,000_ < 0.001, η^2^_900–1,000_ = 0.28. In addition, a three-way interaction was significant in all slow-wave time windows, *F*_600–700_ (4, 124) = 12.62, *p*_600–700_ < 0.001, η^2^_600–700_ = 0.29; *F*_700–800_ (4, 124) = 23.47, *p*_700–800_ < 0.001, *?*^2^_700–800_ = 0.43; *F*_800–900_ (4, 124) = 12.25, *p*_800–900_ < 0.001, η^2^_800–900_ = 0.28; *F*_900–1,000_ (4, 124) = 25.07, *p*_900–1,000_ < 0.001, η^2^_900–1,000_ = 0.45. Further analysis revealed that from 600 to 700 ms, high EOL judgments were higher in mean amplitude than low EOL judgments (all *p* < 0.05) in FR, CL, and CM ROIs. From 700 to 1,000 ms, high EOL judgments were higher in mean amplitude than low EOL judgments (all *p* < 0.05) in FL, FR, CL, CM, and CR ROIs.

### Source Analysis

**FIGURE 4:**
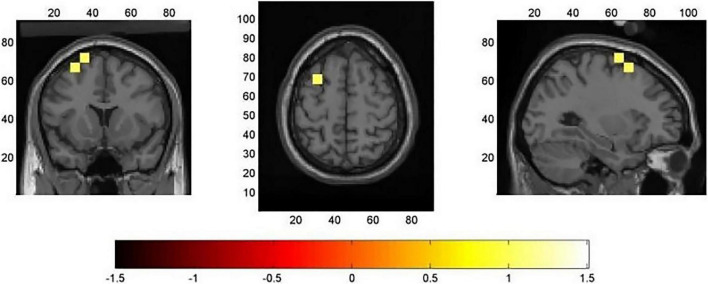
Results of minimum norm estimate (MNE) source analysis in the time window of the N400 component (400–600 ms). The images represent the voxels in which the “low > high easy of learning (EOL) judgment” contrast was significant (*p* < 0.05), and they have been corrected using a cluster permutation test. The significantly activated voxels are indicated by yellow colors.

For the averaged time window between 400 and 600 ms, a significant higher cortical activation for low EOL judgments in contrast to high condition was found in the following cortical areas (see Figure 4): the left superior frontal gyrus [SFG; BA6; *x* = − 18, *y* = 12, *z* = 68, *t*(31) = 2.96, *p* < 0.05] and left middle frontal gyrus [MFG; BA8; *x* = − 30, *y* = 12, *z* = 58, *t*(31) = 2.96, *p* < 0.05].

**FIGURE 5:**
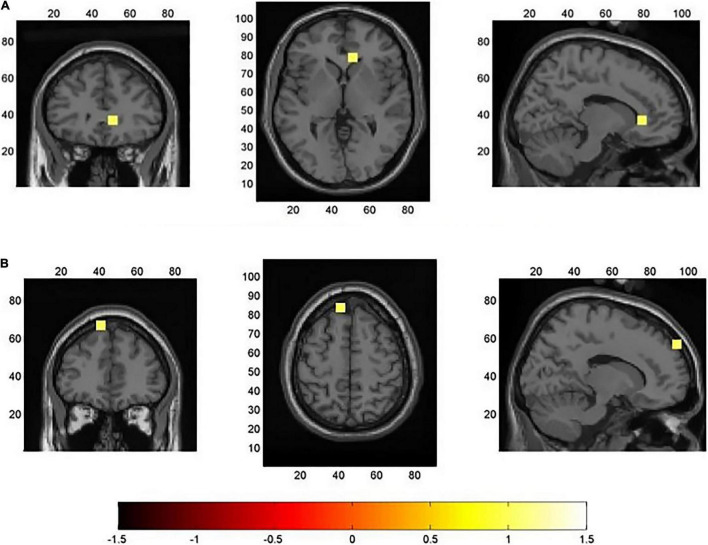
Results of minimum norm estimate (MNE) source analysis in the time window of the slow-wave potentials. **(A)** Source localization result from 600 to 700 ms. **(B)** Source localization result from 700 to 1,000 ms. The images represent the voxels in which the “high > low easy of learning (EOL) judgment” contrast was significant (*p* < 0.05), and they have been corrected using a cluster permutation test. The significantly activated voxels are indicated by yellow colors.

For the averaged time window between 600 and 700 ms (see Figure 5A), we found the cortical activation differences for high EOL judgments in contrast to low condition at the left medial temporal lobe [MTL; BA20, *x* = − 36, *y* = − 38, *z* = − 42, *t*(31) = 1.96, *p* < 0.05] and right ventromedial prefrontal cortex [VMPFC; BA11, *x* = 12, *y* = 30, *z* = 0, *t*(31) = 1.99, *p* < 0.05]. For the averaged time window from 700 to 800, 800–900, and 900–1,000 ms (see Figure 5B), compared to low EOL judgments, cortical activation differences were found for high condition at left DLPFC [BA9, *x* = 12, *y* = 26, *z* = − 4, all *t*(31) > 1.96, all *p* < 0.05] and right DLPFC [BA9, *x* = 12, *y* = 62, *z* = 40, all *t*(31) > 1.96, all *p* < 0.05). The source localization results for each time windows (700–800, 800–900, and 900–1,000 ms) are same, so this study presents the common results in Figure 5B.

## Discussion

In the present study, we compared neural ERP components of different EOL judgments (high, low) using an EOL paradigm, then source localization was conducted to explore the neural basis of corresponding components. Thus, the role of coding fluency cues in the temporal dynamics of EOL judgments was verified.

### Behavior Contribution of Different Easy of Learning Judgments to the Ease-of-Processing Hypothesis

The behavior results showed that the judgment time (RT) of high EOL judgments was shorter than low EOL judgments. The recognition performance was increased with magnitudes. In other words, an item was judged as easy to learn in the EOL phase, and participants would perform better for this item in the recognition test. The result of judgment time in making EOL magnitude is shown that high and low EOL judgments were different types. High EOL judgments are a kind of EOL judgments that require fast encoding, which has a more fluently encoding than low EOL judgments. Thus, it spent a shorter judgment time in the EOL phase. However, low EOL judgments are a type of EOL judgments that need a longer judgment time in the EOL phase, and they acquire few encoding fluency clues. In addition, the recognition performance between high and low EOL judgments also revealed both of which are different types of EOL judgments. In subsequent recognition tests, items previously judged easy to learn in the EOL phase could be successfully recognized, while items judged hard to learn will lower the recognition possibility.

In a previous study, participants would use fluency as clues when they judge how likely the word is to be learned (Jemstedt et al., 2017), which inferred that coding fluency is a factor that influences EOL judgments. The ease-of-learning hypothesis suggested that metacognitive monitoring magnitude was affected by encoding fluency clues (Undorf and Erdfelder, 2011). This study made inferences that coding fluency affected EOL judgment magnitude. In other words, participants gave lower EOL magnitudes to items presented with a higher learning difficulty (hard to learn) as these items had lower coding fluency clues. Participants gave higher EOL magnitudes to items with a lower learning difficulty (easy to learn) as these items had higher coding fluency clues. However, these inferences should be tested in further behavioral research. Besides, other interesting results that need further exploration are that the EOL judgments’ RT was longest at magnitudes 3 and 4. EOL judgments reflect one’s confidence for to be learned items, the highest and lowest EOL judgment expressions of strong confidence that the item will or will not be remembered, which have higher judgment speed. However, when participants hesitate to make EOL judgments, they lack confidence that the item will or will not be remembered, giving a longer judgment time to produce middle ratings (magnitude 3 and magnitude 4). This U-shaped judgment RT phenomenon has been shown in JOL judgment research (Dunlosky et al., 2005), and they noted that making extreme JOL is equivalent to stating extreme confidence in the recall outcome. It would be an underlying interpretation and need further investigation in future behavioral studies.

### The Role of the N400 and Slow-Wave Potentials on Different Easy of Learning Judgments

N400 was induced from both high and low EOL judgments, and the mean amplitude of N400 evoked by low EOL judgments was higher than high EOL judgment ratings. From the perspective of hemispheres, low EOL judgments observed higher mean amplitude at the midline electrodes. It is important to note that Undorf et al. (2020) used word pairs with different associations as fluency cues that investigate ERP components related to JOL. The results showed that the most significant change in the N400 relatedness effect occurred and verified N400 as a processing fluency index on JOL. Our results confirmed that encoding fluency cues affected EOL judgment processing. When encoding an item that lacked fluency, the N400 mean amplitude was stronger and produced low EOL judgment magnitude.

A positive slow wave was observed during EOL judgments in the time window from 600 to 1,000 ms, and high EOL judgments elicited higher amplitude than low EOL judgments, which reflected the metacognitive monitoring process (Skavhaug et al., 2010; Müller et al., 2016). Skavhaug et al. (2010) observed a higher positive slow-wave amplitude of high-magnitude metacognitive monitoring (JOL) than low-magnitude condition at the right frontal region in the late time window (350–700 ms), confirming the impact of frontal slow-wave potentials on metacognitive monitoring. In our study, when compared to low EOL judgments, high EOL judgments elicited a stronger mean amplitude of the positive slow-wave at frontal-central regions from 600 to 1,000 ms. Expressly, our results indicated that the positive slow-wave potentials at frontal-central regions are more likely to be related to metacognition monitoring. The ease-of-processing hypothesis explains that high EOL judgments have stronger positive slow-wave potentials. In other words, when the encoding of EOL is fluent, participants could obtain more encoding fluency cues, thus, producing stronger positive slow-wave and reflecting on high EOL judgments. Consequently, our ERP results of N400 and slow-wave potentials proved that individuals made metacognitive monitoring judgments based on encoding fluency cues.

### The Neural Correlate of Different Easy of Learning Judgments

The results of source localization pointed to the role of encoding fluency cues in EOL judgments. Source localization of the N400 showed that lower EOL judgment magnitude had stronger activation than high EOL conditions in the left SFG and left MFG, suggesting that individuals acquire encoding fluency cues before formal metacognitive monitoring. Support for this comes from Proverbio and De Benedetto (2018), who reported that SFG and MFG are related to encoding activity. In addition, a previous study noted that N400 is a fluency index on JOL and a lower JOL magnitude with a higher N400 amplitude (Undorf et al., 2020). This study used the EOL paradigm that found higher N400 components in low EOL judgment conditions that are originated from higher activation in left SFG and left MFG, and the activation of the above brain regions reflected the process of individuals’ acquiring encoding fluency cues. That is, after the stimulus onset 400–600 ms, coding fluency affected the activation in left SFG and left MFG, and the lower coding fluency induced the higher activation in SFG and MFG, then produced higher N400 amplitude in the scalp. The ease-of-processing hypothesis provided an interpretation that low EOL judgments have stronger SFG and MFG activation. Under the low EOL judgment conditions, items lack coding fluency clues and individuals should try their best to code these items, then eliciting higher activation in SFG and MFG, finally producing higher N400 amplitude in the scalp. Therefore, the N400 amplitude was increased with the activation of SFG and MFG.

The source analysis results in slow-wave potentials are divided into two parts, including 600–700 and 700–1,000 ms (700–800, 800–900, and 900–1,000 ms have the same brain regions).

From 600 to 700 ms time window, this study found more cortical activation at the left MTL and right VMPFC for high EOL judgments than low EOL conditions. It can be seen that a larger amplitude in the FL and CL ROIs was originated from left MTL, and the enlarged amplitude at the FR region results in the right VMPFC. It is reported that MTL is related to encoding in word pairs memory tasks (Rey et al., 2018). A functional magnetic resonance imaging (fMRI) study related to JOL revealed that the magnitude of metacognitive monitoring was linked to VMPFC intensity, and the activation of VMPFC was increased with the magnitude of metacognitive monitoring (Yang et al., 2015). The activation of MTL and VMPFC from 600 to 700 ms gave further evidence that metacognitive monitoring relied on encoding fluency cues required from the previous stage. These results align with the ease-of-processing hypothesis that EOL judgments are mainly based on encoding fluency, making metacognitive monitoring. When it comes to more fluently encoding during EOL judgments, individuals gained more encoding fluency cues and produced high EOL judgments, thus, reflecting more activation linking to encoding and metacognitive monitoring regions.

The source localization for slow-wave potentials (700–1,000 ms) revealed greater activation in DLPFC for high EOL judgments than low EOL judgments, suggesting the metacognitive monitoring process. Furthermore, the slow-wave of high EOL judgment condition from frontal-central ROIs may originate from bilateral DLPFC. Some metacognitive monitoring studies have reported that the potentials of the prefrontal cortex regulated metacognitive monitoring magnitudes (Irak et al., 2019), and this study further confirmed that DLPFC had influenced the magnitude of metacognitive monitoring. Chua et al. (2014) reviewed the effect of DLPFC on metacognitive monitoring magnitude (JOL). In this study, the activation on DLPFC was increased with the magnitude of metacognitive monitoring. In addition, Yang et al. (2015) used the fMRI technique to investigate the neural mechanism in JOL magnitude. The results revealed that JOL magnitude is correlated with VMPFC, posterior cingulate cortex (PCC), DLPFC, and the visual cortex. Thus, our finding observed the impact of VMPFC and DLPFC on EOL judgment magnitude. Under the high EOL judgment conditions, individuals made EOL judgments more fluently than low EOL judgment conditions and induced higher activation in VMPFC and DLPFC, finally produced higher slow-wave amplitude in the scalp.

From the source localization in slow-wave potentials time windows, it is concluded that after the stimulus onset 600–1,000 ms, participants used coding fluency clues to make EOL judgments.

### Important Findings and Future Research Directions

Based on the above analysis, it can be seen that the time processing of EOL judgments had two steps and was affected by encoding fluency. The differences in ERP amplitude and activation intensity of brain regions between high and low EOL judgments reflected the discrepancies in the strengths of encoding fluency cues used in specific time windows. Specifically, we found that N400 had appeared after stimulus onset, which revealed the process of acquiring encoding fluency cues. When items were encoded without fluency during the N400 time window, individuals would produce low EOL judgments, and the brain region with encoding-related activation was strong. After obtaining encoding fluency cues, individuals made EOL judgments around 600–1,000 ms. The judgment magnitude of EOL was increased with encoding fluency cues, thus causing stronger activation of brain regions linking to metacognitive monitoring.

Our results provide behavioral and neurophysiological evidence for the ease-of-processing hypothesis, verify the influence of encoding fluency cues on EOL judgments, and enrich the research on the neural mechanism of metacognitive monitoring. These results should be examined further in future studies using similar tasks. However, there are some limitations to the present study. Firstly, due to the high temporal resolution of ERP technology, the source localization cannot accurately locate the whole brain. Future studies should use the fMRI technique with a high spatial resolution to further confirm and develop the source location results of this study. In addition, non-invasive techniques, such as repeated transcranial magnetic stimulation (rTMS), can be used to validate functional brain regions associated with EOL judgments. Secondly, previous studies have found that metacognitive function deficits in patients with schizophrenia (Francis et al., 2017) acquired brain injury (Lemaitre et al., 2018), and borderline personality disorder (Quattrini et al., 2019). It is essential to investigate the behavioral performances of clinical patients in EOL tasks and the relative brain mechanisms to provide targeted guidance for the metacognitive function recovery of clinical groups. Thirdly, both EOL and JOL judgments are prospective metacognitive monitoring, and they have performed similarity in behavior aspect. Based on the current research results of JOL judgments, this study determined the time course and ERP components that EOL judgments concentrate on. In addition, the ERP analysis finally verified that EOL and JOL judgments both have the process of attaining and utilizing encoding fluency cues. However, there are still differences between them. For example, when compared with EOL judgments, JOL (significantly delayed JOL) additionally has the process of attempted retrieval, individuals using the retrieval fluency to make metacognitive monitoring judgment. While the cues using and judgment magnitudes producing are different in other types of metacognitive monitoring (e.g., FOK and JOC). Therefore, cognitive neuroscience technologies, such as ERP, can be used in future studies to deeply compare the brain mechanisms of different types of metacognitive monitoring and provide strong evidence for the internal separation of metacognitive monitoring.

## Conclusion

The present study confirmed the ease-of-processing hypothesis from behavior and neural correlate aspects by comparing EOL judgment magnitudes in ERP component amplitude and brain activation regions through ERP and source analysis. It is also found that EOL judgments’ processing can be divided into two stages. In the first stage, encoding fluency is obtained as essential cues, and in the second stage, encoding fluency is used to make monitoring judgments.

## Data Availability Statement

The raw data supporting the conclusions of this article will be made available by the authors, without undue reservation.

## Ethics Statement

The studies involving human participants were reviewed and approved by Ethical Review Committee of Hebei Normal University. The patients/participants provided their written informed consent to participate in this study.

## Author Contributions

PC and NJ: writing and editing. Both authors have read and agreed to the published version of the manuscript.

## Conflict of Interest

The authors declare that the research was conducted in the absence of any commercial or financial relationships that could be construed as a potential conflict of interest.

## Publisher’s Note

All claims expressed in this article are solely those of the authors and do not necessarily represent those of their affiliated organizations, or those of the publisher, the editors and the reviewers. Any product that may be evaluated in this article, or claim that may be made by its manufacturer, is not guaranteed or endorsed by the publisher.

## Footnotes

1
http://fieldtrip.fcdonders.nl/

## References

[B1] Baars M. Vink S. van Gog T. de Bruin A. Paas F. (2014). Effects of training self-assessment and using assessment standards on retrospective and prospective monitoring of problem solving. *Learn. Instruct.* 33 92–107. 10.1016/j.learninstruc.2014.04.004

[B2] Beijing Language and Culture University (1986). *Frequency Dictionary of Modern Chinese.* Beijing: Beijing Language and Culture Institute Press.

[B3] Bellon E. Fias W. De Smedt B. (2020). Metacognition across domains: is the association between arithmetic and metacognitive monitoring domain-specific? *PLoS One* 15:e0229932. 10.1371/journal.pone.0229932 32163453 PMC7067420

[B4] Chua E. F. Pergolizzi D. Weintraub R. R. (2014). “The cognitive neuroscience of metamemory monitoring: understanding metamemory processes, subjective levels expressed, and metacognitive accuracy,” in *The Cognitive Neuroscience of Metacognition*, eds Fleming S. M. Frith C. D. (Berlin: Springer), 267–291. 10.1007/978-3-642-45190-4_12

[B5] Chua E. F. Schacter D. L. Sperling R. A. (2009). Neural correlates of metamemory: a comparison of feeling-of-knowing and retrospective confidence judgments. *J. Cogn. Neurosci.* 21 1751–1765. 10.1162/jocn.2009.21123 18823230 PMC2709699

[B6] Cosentino S. Brickman A. M. Griffith E. Habeck C. Cines S. Farrell M. (2015). The right insula contributes to memory awareness in cognitively diverse older adults. *Neuropsychologia* 75 163–169. 10.1016/j.neuropsychologia.2015.05.032 26049091 PMC4546518

[B7] Delorme A. Makeig S. (2004). EEGLAB: an open source toolbox for analysis of single-trial EEG dynamics including independent component analysis. *J. Neurosci. Methods* 134 9–21. 10.1016/j.jneumeth.2003.10.009 15102499

[B8] d’Oleire Uquillas F. Jacobs H. I. Schultz A. P. Hanseeuw B. J. Buckley R. F. Sepulcre J. (2020). Functional and pathological correlates of judgments of learning in cognitively unimpaired older adults. *Cereb. Cortex* 30 1974–1983. 10.1093/cercor/bhz217 31696223 PMC7132950

[B9] Dunlosky J. Serra M. J. Matvey G. Rawson K. A. (2005). Second-order judgments about judgments of learning. *J. Gen. Psychol.* 132 335–346. 10.3200/GENP.132.4.335-346

[B10] Francis M. M. Hummer T. A. Leonhardt B. L. Vohs J. L. Yung M. G. Mehdiyoun N. F. (2017). Association of medial prefrontal resting-state functional connectivity and metacognitive capacity in early phase psychosis. *Psychiatry Res. Neuroimaging* 262 8–14. 10.1016/j.pscychresns.2016.12.014 28208070

[B11] Gaynor A. M. Chua E. F. (2019). Transcranial direct current stimulation over the prefrontal cortex alters encoding and judgments of learning based on fluency. *J. Cogn. Neurosci.* 31 1710–1725. 10.1162/jocn_a_01449 31322469

[B12] Gramfort A. Luessi M. Larson E. Engemann D. A. Strohmeier D. Brodbeck C. (2014). MNE software for processing MEG and EEG data. *Neuroimage* 86 446–460. 10.1016/j.neuroimage.2013.10.027 24161808 PMC3930851

[B13] Greenhouse S. W. Geisser S. (1959). On methods in the analysis of profile data. *Psychometrika* 24 95–112. 10.1007/BF02289823

[B14] Hu X. Liu Z. Chen W. Zheng J. Su N. Wang W. (2017). Individual differences in the accuracy of judgments of learning are related to the gray matter volume and functional connectivity of the left mid-insula. *Front. Hum. Neurosci.* 11:399. 10.3389/fnhum.2017.00399 28824403 PMC5539074

[B15] Irak M. Soylu C. Turan G. (2020). Comparing electrophysiological correlates of judgment of learning and feeling of knowing during face-name recognition. *Cogn. Neuropsychol.* 36 1–22. 10.1080/02643294.2019.1707650 31928316

[B16] Irak M. Soylu C. Turan G. Çapan D. (2019). Neurobiological basis of feeling of knowing in episodic memory. *Cogn. Neurodynamics* 13 239–256. 10.1007/s11571-019-09520-5 31168329 PMC6520417

[B17] Jemstedt A. Kubik V. Jönsson F. U. (2017). What moderates the accuracy of ease of learning judgments? *Metacogn. Learn.* 12 337–355. 10.1007/s11409-017-9172-3

[B18] Jemstedt A. Schwartz B. L. Jönsson F. U. (2018). Ease-of-learning judgments are based on both processing fluency and beliefs. *Memory* 26 807–815. 10.1080/09658211.2017.1410849 29243535

[B19] Jia N. (2012). The progress and prospect of the research on delayed judgment of learning. *J. Psychol. Sci.* 35, 62–69.

[B20] Kelley T. D. McNeely D. A. Serra M. J. Davis T. (2020). Delayed judgments of learning are associated with activation of information from past experiences: a neurobiological examination. *Psychol. Sci.* 32 96–108. 10.1177/0956797620958004 33275057

[B21] Kutas M. Federmeier K. D. (2011). Thirty years and counting: finding meaning in the n400 component of the event-related brain potential (ERP). *Annu. Rev. Psychol.* 62 621–647. 10.1146/annual.psych.093008.131123 20809790 PMC4052444

[B22] Lemaitre A. L. Herbet G. Duffau H. Lafargue G. (2018). Preserved metacognitive ability despite unilateral or bilateral anterior prefrontal resection. *Brain Cogn.* 120 48–57. 10.1016/j.bandc.2017.10.004 29122369

[B23] Lemieux C. L. Collin C. A. Watier N. N. (2019). Gender differences in metacognitive judgments and performance on a goal-directed wayfinding task. *J. Cogn. Psychol.* 31 453–466. 10.1080/20445911.2019.1625905

[B24] Liu C. Chen G. X. Li K. Y. (2019). Time course of delayed judgment of learning. *Psychol. Exploration* 39 232–237.

[B25] Mazziotta J. Toga A. Evans A. Fox P. Lancaster J. Zilles K. (2001). A probabilistic atlas and reference system for the human brain: International Consortium for Brain Mapping (ICBM). *Philos. Trans. R. Soc. London. Series B: Biol. Sci.* 356 1293–1322. 10.1098/rstb.2001.0915 11545704 PMC1088516

[B26] Morales J. Lau H. Fleming S. M. (2018). Domain-general and domain-specific patterns of activity supporting metacognition in human prefrontal cortex. *J. Neurosci.* 38 3534–3546. 10.1523/JNEUROSCI.2360-17.2018 29519851 PMC5895040

[B27] Müller B. C. Tsalas N. R. van Schie H. T. Meinhardt J. Proust J. Sodian B. (2016). Neural correlates of judgments of learning-An ERP study on metacognition. *Brain Res.* 1652 170–177. 10.1016/j.brainres.2016.10.005 27720854

[B28] Nelson T. O. (1996). Gamma is a measure of the accuracy of predicting performance on one item relative to another item, not of the absolute performance on an individual item comments on Schraw. *Appl. Cogn. Psychol.* 10 257–260.

[B29] Pernet C. R. Latinus M. Nichols T. E. Rousselet G. A. (2015). Cluster-based computational methods for mass univariate analyses of event-related brain potentials/fields: a simulation study. *J. Neurosci. Methods* 250 85–93. 10.1016/j.jneumeth.2014.08.003 25128255 PMC4510917

[B30] Picton T. W. Bentin S. Berg P. Donchin E. Hillyard S. A. Johnson R. (2000). Guidelines for using human event-related potentials to study cognition: recording standards and publication criteria. *Psychophysiology* 37 127–152. 10.1111/1469-8986.3720127 10731765

[B31] Proverbio A. M. De Benedetto F. (2018). Auditory enhancement of visual memory encoding is driven by emotional content of the auditory material and mediated by superior frontal cortex. *Biol. Psychol.* 132 164–175. 10.1016/j.biopsycho.2017.12.003 29292233

[B32] Quattrini G. Pini L. Pievani M. Magni L. R. Lanfredi M. Ferrari C. (2019). Abnormalities in functional connectivity in borderline personality disorder: correlations with metacognition and emotion dysregulation. *Psychiatry Res.: Neuroimaging* 283 118–124. 10.1016/j.pscychresns.2018.12.010 30591402

[B33] Rey H. G. De Falco E. Ison M. J. Valentin A. Alarcon G. Selway R. (2018). Encoding of long-term associations through neural unitization in the human medial temporal lobe. *Nat. Commun.* 9:4372. 10.1038/s41467-018-06870-2 30348996 PMC6197188

[B34] Rosen H. J. Alcantar O. Zakrzewski J. Shimamura A. P. Neuhaus J. Miller B. L. (2014). Metacognition in the behavioral variant of frontotemporal dementia and Alzheimer’s disease. *Neuropsychology* 28 436–447. 10.1037/neu0000012 24548124 PMC4085356

[B35] Rösler F. Heil M. Röder B. (1997). Slow negative brain potentials as reflections of specific modular resources of cognition. *Biol. Psychol.* 45 109–141. 10.1016/S0301-0511(96)05225-8 9083647

[B36] Skavhaug I. M. Wilding E. L. Donaldson D. I. (2010). Judgments of learning do not reduce to memory encoding operations: event-related potential evidence for distinct metacognitive processes. *Brain Res.* 1318 87–95. 10.1016/j.brainres.2009.11.047 19968975

[B37] Tabachnick B. F. L. Fidell L. S. (2013). *Using Multivariate Statistics.* 6th Edn. London: Pearson, 10.1037/022267

[B38] Tsalas N. R. Müller B. C. Meinhardt J. Proust J. Paulus M. Sodian B. (2018). An ERP study on metacognitive monitoring processes in children. *Brain Res.* 1695 84–90. 10.1016/j.brainres.2018.05.041 29852136

[B39] Undorf M. Amaefule C. O. Kamp S. M. (2020). The neurocognitive basis of metamemory: using the N400 to study the contribution of fluency to judgments of learning. *Neurobiol. Learn. Mem.* 169:107176. 10.1016/j.nlm.2020.107176 32001337

[B40] Undorf M. Erdfelder E. (2011). Judgments of learning reflect encoding fluency: conclusive evidence for the ease-of-processing hypothesis. *J. Exp. Psychol. Learn. Memory Cogn.* 37 1264–1269. 10.1037/a0023719 21574748

[B41] Vaccaro A. G. Fleming S. M. (2018). Thinking about thinking: a coordinate-based meta-analysis of neuroimaging studies of metacognitive judgements. *Brain Neurosci. Adv.* 2:2398212818810591. 10.1177/2398212818810591 30542659 PMC6238228

[B42] Voloshyna V. Jonsson F. (2012). “Do different metamemory judgments share the same underlying cognitive processes?,” in *Proceedings of the 5th International Conference on Cognitive Science*, (Kaliningrad). 10.1098/rspb.2017.2035

[B43] Wu H. (2012). The influence of material factors on easy of learning judgment and its accuracy. *J. Guizhou Normal University* 30 22–25. 10.16614/j.cnki.issn1004-5570.2012.05.003

[B44] Yang H. Cai Y. Liu Q. Zhao X. Wang Q. Chen C. (2015). Differential neural correlates underlie judgment of learning and subsequent memory performance. *Front. Psychol.* 6:1699. 10.3389/fpsyg.2015.01699 26617540 PMC4637415

[B45] Yu L. Schack T. Koester D. (2021). Online movement correction in response to the unexpectedly perturbed initial or final action goals: an ERP and sLORETA study. *Brain Sci.* 11:641. 10.3390/brainsci11050641 34063437 PMC8156469

