# Supplementary material for: An Event-Related Potential Study on Differences Between Higher and Lower Easy of Learning Judgments: Evidence for the Ease-of-Processing Hypothesis
Source: Front Psychol. 2022 Mar 18;13:779907. doi: 10.3389/fpsyg.2022.779907 (PMC8972125; doi:10.3389/fpsyg.2022.779907)
Supplement: Supplementary file 1 [file Data_Sheet_1.docx]

Supplementary Material

# Supplementary Data

**Supplementary** **Table 1 Materials using in experiment**

| Difficult word pairs | Cue word | Target word | *N* | minimum | maximum | mean | *SD* |
| --- | --- | --- | --- | --- | --- | --- | --- |
| 1 | 枝叶 | 抹布 | 39 | 1.00 | 6.00 | 1.05 | 0.32 |
| 2 | 盖头 | 佣金 | 39 | 1.00 | 6.00 | 1.21 | 1.00 |
| 3 | 豆汁 | 旧账 | 39 | 1.00 | 6.00 | 1.21 | 1.00 |
| 4 | 油脂 | 名牌 | 39 | 1.00 | 6.00 | 1.23 | 1.01 |
| 5 | 毛孩 | 白城 | 39 | 1.00 | 6.00 | 1.23 | 0.99 |
| 6 | 飞贼 | 沙坑 | 39 | 1.00 | 6.00 | 1.28 | 1.05 |
| 7 | 炕头 | 车组 | 39 | 1.00 | 6.00 | 1.33 | 1.34 |
| 8 | 社论 | 波长 | 39 | 1.00 | 6.00 | 1.36 | 1.37 |
| 9 | 圣药 | 黑土 | 39 | 1.00 | 6.00 | 1.38 | 1.37 |
| 10 | 钩子 | 使团 | 39 | 1.00 | 6.00 | 1.41 | 1.37 |
| 11 | 香油 | 酒杯 | 39 | 1.00 | 6.00 | 1.44 | 1.43 |
| 12 | 期货 | 衣柜 | 39 | 1.00 | 6.00 | 1.46 | 1.39 |
| 13 | 橙汁 | 票房 | 39 | 1.00 | 6.00 | 1.46 | 1.43 |
| 14 | 农妇 | 亲王 | 39 | 1.00 | 6.00 | 1.49 | 1.62 |
| 15 | 蛇头 | 卧房 | 39 | 1.00 | 6.00 | 1.49 | 1.62 |
| 16 | 斧头 | 经书 | 39 | 1.00 | 6.00 | 1.51 | 1.64 |
| 17 | 电场 | 眼线 | 39 | 1.00 | 6.00 | 1.51 | 1.64 |
| 18 | 木柴 | 球门 | 39 | 1.00 | 6.00 | 1.51 | 1.64 |
| 19 | 眉目 | 示例 | 39 | 1.00 | 6.00 | 1.54 | 1.64 |
| 20 | 颈椎 | 路费 | 39 | 1.00 | 6.00 | 1.59 | 1.68 |
| 21 | 冰点 | 光束 | 39 | 1.00 | 6.00 | 1.59 | 1.68 |
| 22 | 头盔 | 雀斑 | 39 | 1.00 | 6.00 | 1.64 | 1.84 |
| 23 | 村姑 | 快车 | 39 | 1.00 | 6.00 | 1.67 | 1.85 |
| 24 | 皮具 | 本金 | 39 | 1.00 | 6.00 | 1.69 | 1.76 |
| 25 | 函件 | 推子 | 39 | 1.00 | 6.00 | 1.72 | 1.86 |
| 26 | 体坛 | 天堑 | 39 | 1.00 | 6.00 | 1.72 | 1.86 |
| 27 | 海鸥 | 糯米 | 39 | 1.00 | 6.00 | 1.72 | 1.86 |
| 28 | 肉鸡 | 事主 | 39 | 1.00 | 6.00 | 1.72 | 1.86 |
| 29 | 古物 | 淡水 | 39 | 1.00 | 6.00 | 1.72 | 1.85 |
| 30 | 图纸 | 平原 | 39 | 1.00 | 6.00 | 1.79 | 2.03 |
| 31 | 泥沼 | 群山 | 39 | 1.00 | 6.00 | 1.79 | 1.89 |
| 32 | 巫医 | 尖兵 | 39 | 1.00 | 6.00 | 1.82 | 2.04 |
| 33 | 北极 | 娘子 | 39 | 1.00 | 6.00 | 1.85 | 2.02 |
| 34 | 旱地 | 庙宇 | 39 | 1.00 | 6.00 | 1.85 | 2.03 |
| 35 | 大料 | 名师 | 39 | 1.00 | 6.00 | 1.85 | 2.03 |
| 36 | 水珠 | 馅饼 | 39 | 1.00 | 6.00 | 1.85 | 2.03 |
| 37 | 沙盘 | 酒宴 | 39 | 1.00 | 6.00 | 1.85 | 2.03 |
| 38 | 水酒 | 床板 | 39 | 1.00 | 6.00 | 1.85 | 1.98 |
| 39 | 燕雀 | 淀粉 | 39 | 1.00 | 6.00 | 1.87 | 2.03 |
| 40 | 木船 | 囚徒 | 39 | 1.00 | 6.00 | 1.87 | 2.04 |
| 41 | 猩猩 | 边境 | 39 | 1.00 | 6.00 | 1.90 | 2.04 |
| 42 | 烙印 | 座机 | 39 | 1.00 | 6.00 | 1.90 | 2.09 |
| 43 | 孔洞 | 工笔 | 39 | 1.00 | 6.00 | 1.90 | 2.05 |
| 44 | 林地 | 印泥 | 39 | 1.00 | 6.00 | 1.92 | 2.09 |
| 45 | 香菇 | 器官 | 39 | 1.00 | 6.00 | 1.95 | 2.09 |
| 46 | 草木 | 城池 | 39 | 1.00 | 6.00 | 1.97 | 2.19 |
| 47 | 总务 | 行当 | 39 | 1.00 | 6.00 | 2.00 | 2.19 |
| 48 | 沙尘 | 枝节 | 39 | 1.00 | 6.00 | 2.00 | 2.19 |
| 49 | 道人 | 石柱 | 39 | 1.00 | 6.00 | 2.03 | 2.18 |
| 50 | 凭证 | 足金 | 39 | 1.00 | 6.00 | 2.03 | 2.18 |
| 51 | 民房 | 弹弓 | 39 | 1.00 | 6.00 | 2.03 | 2.15 |
| 52 | 灯泡 | 风筝 | 39 | 1.00 | 6.00 | 2.08 | 2.21 |
| 53 | 花丛 | 烟斗 | 39 | 1.00 | 6.00 | 2.13 | 2.33 |
| 54 | 字条 | 西语 | 39 | 1.00 | 6.00 | 2.18 | 2.32 |
| 55 | 姑父 | 贝勒 | 39 | 1.00 | 6.00 | 2.21 | 2.33 |
| 56 | 草场 | 连杆 | 39 | 1.00 | 6.00 | 2.23 | 2.37 |
| 57 | 国法 | 外宾 | 39 | 1.00 | 6.00 | 2.26 | 2.24 |
| 58 | 天梯 | 砧板 | 39 | 1.00 | 6.00 | 2.33 | 2.44 |
| 59 | 汗衫 | 果皮 | 39 | 1.00 | 6.00 | 2.36 | 2.52 |
| 60 | 社员 | 家业 | 39 | 1.00 | 6.00 | 2.44 | 2.47 |
| 61 | 样片 | 西药 | 39 | 1.00 | 6.00 | 2.51 | 2.52 |
| 62 | 木棍 | 桌布 | 39 | 1.00 | 6.00 | 2.51 | 2.52 |
| 63 | 礼金 | 古玩 | 39 | 1.00 | 6.00 | 2.54 | 2.53 |
| 64 | 铜币 | 戏院 | 39 | 1.00 | 6.00 | 2.54 | 2.53 |
| 65 | 皮球 | 废纸 | 39 | 1.00 | 6.00 | 2.56 | 2.55 |
| 66 | 水桶 | 银杏 | 39 | 1.00 | 6.00 | 2.67 | 2.66 |
| 67 | 加法 | 街区 | 39 | 1.00 | 6.00 | 2.67 | 2.66 |
| 68 | 扫把 | 陈设 | 39 | 1.00 | 6.00 | 2.77 | 2.71 |
| 69 | 佛爷 | 捕头 | 39 | 1.00 | 6.00 | 2.82 | 2.71 |
| 70 | 光标 | 字形 | 39 | 1.00 | 6.00 | 2.82 | 2.71 |
| Easy word pairs | Cue word | Target word | *N* | minimum | maximum | mean | *SD* |
| 71 | 织女 | 牛郎 | 39 | 5.00 | 6.00 | 5.88 | 0.45 |
| 72 | 黑板 | 粉笔 | 39 | 5.00 | 6.00 | 5.84 | 0.43 |
| 73 | 花草 | 树木 | 39 | 5.00 | 6.00 | 5.81 | 0.45 |
| 74 | 狂风 | 暴雨 | 39 | 5.00 | 6.00 | 5.81 | 0.55 |
| 75 | 孔子 | 论语 | 39 | 3.00 | 6.00 | 5.81 | 0.70 |
| 76 | 枪支 | 弹药 | 39 | 6.00 | 6.00 | 5.77 | 0.43 |
| 77 | 墨水 | 钢笔 | 39 | 3.00 | 6.00 | 5.65 | 0.92 |
| 78 | 海湾 | 港口 | 39 | 4.00 | 6.00 | 5.65 | 0.75 |
| 79 | 羊水 | 产妇 | 39 | 4.00 | 6.00 | 5.63 | 0.72 |
| 80 | 签证 | 护照 | 39 | 4.00 | 6.00 | 5.56 | 0.83 |
| 81 | 哑巴 | 手语 | 39 | 3.00 | 6.00 | 5.49 | 0.96 |
| 82 | 金银 | 财宝 | 39 | 3.00 | 6.00 | 5.49 | 0.88 |
| 83 | 插座 | 电线 | 39 | 4.00 | 6.00 | 5.42 | 0.76 |
| 84 | 罂粟 | 鸦片 | 39 | 3.00 | 6.00 | 5.40 | 1.00 |
| 85 | 纱布 | 绷带 | 39 | 4.00 | 6.00 | 5.40 | 0.88 |
| 86 | 秋风 | 落叶 | 39 | 3.00 | 6.00 | 5.37 | 1.02 |
| 87 | 大堂 | 前台 | 39 | 3.00 | 6.00 | 5.33 | 1.02 |
| 88 | 亲朋 | 挚友 | 39 | 4.00 | 6.00 | 5.33 | 0.89 |
| 89 | 油漆 | 刷子 | 39 | 4.00 | 6.00 | 5.33 | 0.84 |
| 90 | 饭碗 | 餐具 | 39 | 3.00 | 6.00 | 5.30 | 0.94 |
| 91 | 钟表 | 时针 | 39 | 4.00 | 6.00 | 5.28 | 1.05 |
| 92 | 书桌 | 课本 | 39 | 4.00 | 6.00 | 5.26 | 0.90 |
| 93 | 存款 | 账户 | 39 | 3.00 | 6.00 | 5.23 | 1.088 |
| 94 | 炉子 | 煤炭 | 39 | 4.00 | 6.00 | 5.23 | 0.87 |
| 95 | 房顶 | 屋檐 | 39 | 4.00 | 6.00 | 5.19 | 0.98 |
| 96 | 邮局 | 信箱 | 39 | 3.00 | 6.00 | 5.19 | 1.26 |
| 97 | 钱夹 | 纸币 | 39 | 3.00 | 6.00 | 5.12 | 1.07 |
| 98 | 香皂 | 泡沫 | 39 | 4.00 | 6.00 | 5.0930 | 1.09 |
| 99 | 饰品 | 耳环 | 39 | 3.00 | 6.00 | 5.05 | 1.09 |
| 100 | 拖把 | 抹布 | 39 | 3.00 | 6.00 | 5.02 | 1.28 |
| 101 | 吊瓶 | 针头 | 39 | 3.00 | 6.00 | 5.00 | 1.21 |
| 102 | 饮品 | 吸管 | 39 | 3.00 | 6.00 | 4.98 | 1.26 |
| 103 | 遗体 | 骨灰 | 39 | 2.00 | 6.00 | 4.91 | 1.39 |
| 104 | 辫子 | 马尾 | 39 | 3.00 | 6.00 | 4.91 | 1.21 |
| 105 | 劫匪 | 凶器 | 39 | 2.00 | 6.00 | 4.88 | 1.33 |
| 106 | 跳蚤 | 虱子 | 39 | 2.00 | 6.00 | 4.88 | 1.33 |
| 107 | 尼姑 | 道士 | 39 | 1.00 | 6.00 | 4.86 | 1.36 |
| 108 | 班车 | 巴士 | 39 | 2.00 | 6.00 | 4.81 | 1.28 |
| 109 | 布料 | 丝绸 | 39 | 2.00 | 6.00 | 4.79 | 1.23 |
| 110 | 皇上 | 太监 | 39 | 3.00 | 6.00 | 4.77 | 1.31 |
| 111 | 皮筋 | 发夹 | 39 | 1.00 | 6.00 | 4.74 | 1.72 |
| 112 | 沙子 | 水泥 | 39 | 1.00 | 6.00 | 4.74 | 1.42 |
| 113 | 木材 | 斧头 | 39 | 1.00 | 6.00 | 4.72 | 1.47 |
| 114 | 逃犯 | 手铐 | 39 | 2.00 | 6.00 | 4.72 | 1.49 |
| 115 | 航线 | 灯塔 | 39 | 1.00 | 6.00 | 4.70 | 1.70 |
| 116 | 小溪 | 泉水 | 39 | 2.00 | 6.00 | 4.70 | 1.32 |
| 117 | 兵营 | 主帅 | 39 | 2.00 | 6.00 | 4.67 | 1.41 |
| 118 | 化石 | 标本 | 39 | 1.00 | 6.00 | 4.67 | 1.55 |
| 119 | 酒会 | 来宾 | 39 | 1.00 | 6.00 | 4.65 | 1.38 |
| 120 | 拉丁 | 舞伴 | 39 | 2.00 | 6.00 | 4.65 | 1.46 |
| 121 | 明火 | 月夜 | 39 | 2.00 | 6.00 | 4.65 | 1.46 |
| 122 | 机车 | 油门 | 39 | 2.00 | 6.00 | 4.65 | 1.38 |
| 123 | 老太 | 拐杖 | 39 | 3.00 | 6.00 | 4.60 | 1.38 |
| 124 | 尾气 | 车灯 | 39 | 2.00 | 6.00 | 4.58 | 1.35 |
| 125 | 文具 | 画笔 | 39 | 2.00 | 6.00 | 4.47 | 1.53 |

# Supplementary Tables

**Supplementary** **Table 2.** Performance measures of all participants at different EOL judgments magnitudes（*M*±*SD*）

|  | 1 | 2 | 3 | 4 | 5 | 6 |
| --- | --- | --- | --- | --- | --- | --- |
| EOL judgment time (RT, ms) | 1898±81 | 2003±98 | 2221±90 | 2122±92 | 1894±80 | 1648±67 |
| Percentage across EOL magnitude | 0.17±0.02 | 0.25±0.01 | 0.15±0.01 | 0.11±0.01 | 0.17±0.02 | 0.19±0.02 |
| Recognition performance | 0.66±0.04 | 0.68±0.04 | 0.70±0.04 | 0.81±0.03 | 0.87±0.02 | 0.90±0.02 |

**Supplementary** **Table 3.** The EOL judgment time (RT) and recognition performance between high EOL and low EOL judgments (*M*±*SD*)*.*

|  | Low EOL | High EOL |
| --- | --- | --- |
| EOL judgment time (RT, ms) | 2042±532 | 1778±420 |
| Recognition performance | 0.67±0.19 | 0.85±0.13 |

**Supplementary** **Table 4.** ERP results of 2 (condition: high, low) × 3 (region: frontal, central, parietal) × 3 (hemisphere: left, middle, right) repeated-measured ANOVA

|  | 400-600ms | 600-700ms | 700-800ms | 800-900ms | 900-1000ms |
| --- | --- | --- | --- | --- | --- |
| condition | *F*(1, 31) = 13.22, *p* = 0.001, *ƞ*^2^ = 0.30 | *F*(1, 31) =16.29, *p* = 0.000, *ƞ*^2^ = 0.34 | *F*(1, 31) = 12.10, *p* = 0.002, *ƞ*^2^ = 0.28 | *F*(1, 31) = 16.44, *p* = 0.000, *ƞ*^2^ = 0.35 | *F*(1, 31) = 11.80, *p* = 0.002, *ƞ*^2^ = 0.45 |
| condition × region |  | *F*(2, 62) = 4.32, *p* = 0.017, *ƞ*^2^ = 0.12 | *F*(2, 62) = 4.83, *p* = 0.011, *ƞ*^2^ = 0.33 | *F*(2, 62) = 4.31, *p* = 0.018, *ƞ*^2^ = 0.12 | *F*(2, 62) = 4.82, *p* = 0.011, *ƞ*^2^ = 0.14 |
| condition × hemisphere | *F*(2, 62) = 5.06, *p*=0.009, *ƞ*^2^ = 0.14 | *F*(2, 62) = 5.39, *p* = 0.007, *ƞ*^2^ = 0.18 | *F*(2, 62) = 14.98, *p* = 0.000, *ƞ*^2^ = 0.27 | *F*(2, 62) = 6.71, *p* = 0.002, *ƞ*^2^ = 0.18 | *F*(2, 62) = 15.63, *p* = 0.000, *ƞ*^2^ = 0.34 |
| condition × region × hemisphere |  | *F*(4, 124) = 12.62, *p* = 0.000, *ƞ*^2^ = 0.29 | *F*(4, 124) = 23.47, *p* = 0.000, *ƞ*^2^ = 0.43 | *F*(4, 124) =12.25, *p* = 0.000, *ƞ*^2^ = 0.28 | *F*(4, 124) = 25.07, *p* = 0.000, *ƞ*^2^ = 0.45 |

**Supplementary** **Table 5.** Word frequency independent sample test of easy and hard word pairs between cue words and target words(*M*±*SD)*

|  | type | n | word frequency | | *F* | *p* |
| --- | --- | --- | --- | --- | --- | --- |
|  |  |  | *M* | *SD* |  |  |
| Cue words | Hard word pairs | 70 | 1.72 | 1.95 | 2.18 | 0.361 |
|  | Easy word pairs | 55 | 2.01 | 1.51 |  |  |
| Target words | Hard word pairs | 70 | 2.22 | 2.55 | 2.649 | 0.162 |
|  | Easy word pairs | 55 | 2.82 | 2.11 |  |  |

**Supplementary** **Table 6.** Stroke number of Chinese characters independent sample test of easy and hard word pairs between cue words and target words (*M*±*SD)*

|  | type | n | Stroke number | | *F* | *p* |
| --- | --- | --- | --- | --- | --- | --- |
|  |  |  | *M* | *SD* |  |  |
| Cue words | Hard word pairs | 70 | 14.84 | 2.88 | 12.18 | 0.54 |
|  | Easy word pairs | 55 | 15.24 | 4.05 |  |  |
| Target words | Hard word pairs | 70 | 15.21 | 3.00 | 8.11 | 0.93 |
|  | Easy word pairs | 55 | 15.27 | 4.15 |  |  |
